# Chances and challenges of a long-term data repository in multiple sclerosis: 20th birthday of the German MS registry

**DOI:** 10.1038/s41598-021-92722-x

**Published:** 2021-06-25

**Authors:** Lisa-Marie Ohle, David Ellenberger, Peter Flachenecker, Tim Friede, Judith Haas, Kerstin Hellwig, Tina Parciak, Clemens Warnke, Friedemann Paul, Uwe K. Zettl, Alexander Stahmann

**Affiliations:** 1 MS Forschungs- und Projektentwicklungs-gGmbH (MS Research and Projectdevelopment gGmbH [MSFP]), Krausenstr 50, 30171 Hannover, Germany; 2 Neurological Rehabilitation Center Quellenhof, Bad Wildbad, Germany; 3 Department of Medical Statistics, University Medical Center Göttingen, Göttingen, Germany; 4 Deutsche Multiple Sklerose Gesellschaft, Bundesverband e.V. (German Multiple Sclerosis Society, Federal Association), Hannover, Germany; 5 Department of Neurology, Katholisches Klinikum, St. Joseph Hospital, Ruhr University Bochum, Bochum, Germany; 6 Department of Medical Informatics, University Medical Center Göttingen, Göttingen, Germany; 7 Department of Neurology, Medical Faculty, University Hospital of Cologne, Cologne, Germany; 8 Experimental and Clinical Research Center and NeuroCure Clinical Research Center, Max Delbrueck Center for Molecular Medicine and Charité – Universitätsmedizin Berlin, Berlin, Germany; 9 Department of Neurology, Neuroimmunological Section, University Medical Center Rostock, Rostock, Germany

**Keywords:** Health care, Multiple sclerosis

## Abstract

In 2001, the German Multiple Sclerosis Society, facing lack of data, founded the German MS Registry (GMSR) as a long-term data repository for MS healthcare research. By the establishment of a network of participating neurological centres of different healthcare sectors across Germany, GMSR provides observational real-world data on long-term disease progression, sociodemographic factors, treatment and the healthcare status of people with MS. This paper aims to illustrate the framework of the GMSR. Structure, design and data quality processes as well as collaborations of the GMSR are presented. The registry’s dataset, status and results are discussed. As of 08 January 2021, 187 centres from different healthcare sectors participate in the GMSR. Following its infrastructure and dataset specification upgrades in 2014, more than 196,000 visits have been recorded relating to more than 33,000 persons with MS (PwMS). The GMSR enables monitoring of PwMS in Germany, supports scientific research projects, and collaborates with national and international MS data repositories and initiatives. With its recent pharmacovigilance extension, it aligns with EMA recommendations and helps to ensure early detection of therapy-related safety signals.

## Introduction

Multiple sclerosis (MS) is the most frequent immune mediated disease of the central nervous system and the most common cause of non-traumatic disability among young adults^1,2^. In 2015, the cumulative incidence of MS in Germany was estimated at 18 new cases per 100,000 persons per year using public healthcare insurance data^2^. The yearly prevalence of MS diagnoses has steadily increased but there are still uncertainties concerning its aetiology^3^. The lack of legal reporting requirements for diagnosed MS cases and difficulties involved in accessing routine medical data impede access to reliable information on MS prevalence and incidence, as well as healthcare standards for persons with MS (PwMS) in Germany. Insufficient interoperability of different electronic healthcare record systems is another reason for the lack of a population-based database. A nationwide MS registry with representative coverage of PwMS would offer the opportunity to monitor long-term disease epidemiology and the health of PwMS. Furthermore, a national MS registry can contribute to data on disease costs, cost–benefit-ratios, quality of healthcare, and patient-reported outcomes (PRO), which is important to the evaluation of PwMS’s quality of life and to regulatory decisions^4^.

Treatment guidelines for chronic diseases like MS are often established based upon limited evidence regarding the real-world efficacy and safety of disease modifying treatments (DMT)^5,6^. Additionally, long-term therapeutic effects beyond the duration of phase III clinical trials, and treatment strategy studies are frequently unavailable^7^. Considering the rapid development of DMT and the significant role of the diverse disease characteristics and patient responses to and preferences in treatment, the documentation of complex individual treatment profiles is becoming increasingly important for personalised treatment decisions that take into account individual disease courses and patient preferences^7–9^.

Considering several observational studies indicating that early initiation and continuous use of highly effective DMT may reduce risks of long-term disability progression, there is a need for predictive tools to identify the most beneficial treatment strategies^9,10^. Regarding these needs, registries can provide real-world data to support physicians and PwMS in treatment decisions. Recent incidents of serious adverse events, like endocarditis^11^, or multifocal leukoencephalopathy^12^ in PwMS on DMT highlighted the importance of standardised safety data collection under real-world conditions^13^. In 2017, the European Medicines Agency initiated a workshop bringing together regulatory bodies, market authorisation holders, academics, and registry custodians to discuss the extended use of MS registries for post-authorisation safety monitoring^14^.

This article aims to show the framework and current setup of the German MS registry (GMSR), including its methodology, technological infrastructure, and network as a reliable nationwide long-term data repository and resource for registry-based trials.

## Materials and methods

This article describes the governance, funding, and design of the GMSR. Its history, data collection processes, description of datasets and data quality control mechanisms are outlined, and its research projects presented. Descriptive analysis outlining the status of the GMSR was carried out using R Stat 4.0 (R Foundation, Vienna, Austria). Data for descriptive analyses was extracted 8 January 2021. Data records that were locked due to e.g. documentation errors and records with pending queries were excluded from analysis. Graphical representations include histograms for expanded disability status scale (EDSS) distributions, scatterplots of patient demographics, and clinical characteristics at entry of the GMSR, including two-dimensional density estimates, shown as contour plots (heat maps), and line charts of patients and visit frequency.

## Results

### The GMSR’s objectives

The GMSR aims to provide a reliable long-term data repository for transparency in epidemiology, demographics, disease characteristics, healthcare access and quality of care of PwMS in Germany, and may be used to monitor improvements of healthcare^15^. It also implements and supports scientific research projects such as registry-based (randomised) controlled trials and collaborations with other (inter-)national repositories in a modular fashion^16^. One of its emerging objectives is the collection and reporting of real-world safety data on disease modifying drugs.

### The GMSR’s design

**Figure 1:**
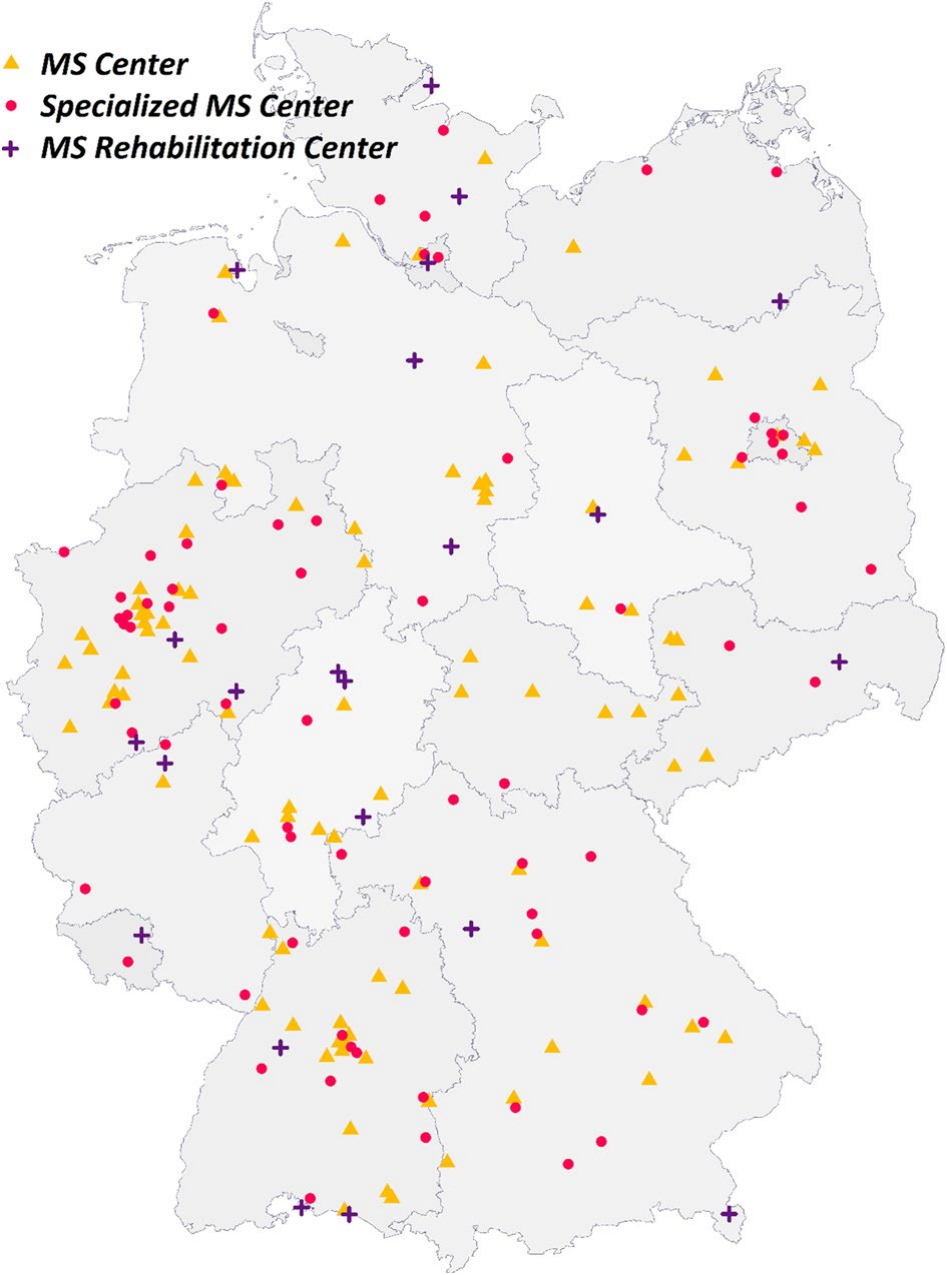
Distribution of 187 MS centres participating in the GMSR across Germany. These include 70 specialised MS centres (red circle), 95 MS centres (orange triangle), and 22 MS rehabilitation centres (purple cross). *GMSR* German Multiple Sclerosis registry. The map was created with R 4.0 based on data from gadm.org.

The GMSR established a network of participating centres across different healthcare sectors in Germany (see Fig. 1). Centres that were accredited ‘MS Centre’, ‘Specialised MS Centre’, or ‘MS Rehabilitation Centre’ by the German MS Society participate in the GMSR documentation. Certificates are awarded to university clinics, acute care clinics, rehabilitation clinics, MS outpatient clinics and resident neurologists complying with specific criteria. Compliance is re-evaluated every 2 years^17^. With this certificate, each centre commits itself to recruiting PwMS and collecting data for the GMSR. Physicians and other medical staff carry out patient enrolment and data collection for the registry during routine examinations. After the provision of written informed consent by adult PwMS (see Table 1), previous medical data can be collected, and prospective data collection starts and is carried out until the patient’s death or withdrawal of informed consent.

**Table 1:** Inclusion and exclusion criteria of the GMSR.

Inclusion criteria	Exclusion criteria
Age ≥ 18 years	Indefinable MS disease type (according to McDonald or Poser criteria) at date of inclusion
Written informed consent provided	Inability to give informed consent
Diagnosed MS (according to the applicable McDonald or Poser criteria) with definable disease type or clinically isolated syndrome (CIS)	
Primary residence in Germany	

*CIS* clinically isolated syndrome, *MS* multiple sclerosis.

Members of the medical staff enter data into a web-based electronic data capture (EDC) system. This system allows a single patient’s data to be reported by multiple centres. To avoid duplicate patient entries, patient-identifying data (first name, last name, sex, date of birth) is entered into the registry at enrolment and used to generate a unique pseudonym. A trusted third party stores these pseudonyms and the corresponding identifying data separated from the registry data. To guarantee patient privacy, only de-identified medical data is stored in the registry’s database and used for analysis.

### Data collection

In 2005, the registry started regular operations and has been continuously improved since then. At first, data was collected through the ‘MSDS Klinik/Praxis’ software, which had been locally installed in centres. Encoded datasets stored digitally were transmitted quarterly to the MSFP-gGmbH (MSFP) for quality checks and analysis. Since 2016, it is mandatory to document via the standardised web-based platform and device-independent EDC-system ‘secuTrial’ and documentation through MSDS has been discontinued. The web-based EDC-system is compliant with established tools and concepts of the Technology and Methods Platform for Networked Medical Research e.V. (TMF) and is certified for conducting clinical trials (GCP, GAMP5, FDA 21 CFR Part 11). PRO can be integrated to the system through patients uploading information themselves through an app or web browser. Data entry happens directly through interfaces of the EDC system.

### Governance of the GMSR

The GMSR is operated by the MSFP, a non-profit organisation founded in 2001 as a subsidiary of the German Multiple Sclerosis Foundation to develop and manage the registry on behalf of the German Multiple Sclerosis Society. The governance of the GMSR includes a scientific advisory group of clinicians and representatives of MS centres as well as methodology experts (of medical statistics, epidemiology and medical informatics), that approves and oversees scientific projects and research activities^18^.

### Ethical considerations/registration

The GMSR was first approved by the ethics committee of the Julius-Maximilians-University of Würzburg (number of vote 142/12). After switching to a web-based documentation system, it received further ratification from ethical committees such as the Thuringia state chamber of physicians and other universities.

Before inclusion to the MS registry, patients are informed about its conduct by medical staff and must provide written informed consent before data collection can commence. Within this consent, patients can allow their data to be used for further research activities. Data collection, storage and analysis are conducted according to current European and national legislations for data protection.

The GMSR is registered at the German registry of clinical trials with registration number DRKS00011257, and at the European Network of Centres for Pharmacoepidemiology and Pharmacovigilance with reference number ENCEPP/DSPP/21378.

### History of GMSR

The history of the GMSR is described in Supplementary Document 1.

### Dataset

In 2014, the GMSR’s dataset was revised to fulfil the increased requirement for harmonization and comparability of data of different data sources. As a result, the dataset is a combination of datasets of the German Multiple Sclerosis Society and the German Competence Network Multiple Sclerosis. The current minimal dataset (see Supplementary Table 1) contains information on disease onsets, diagnoses, MS disease courses^19,20^, disabilities (as measured in EDSS and MS Functional Composite as well by MS Symptoms), disease activity, MS therapy and socio-economic statuses^21^. In 2019, the GMSR dataset was extended by a pharmacovigilance module to include documentation on medical history, body mass index, adverse events and pregnancies (see Table 2). This documentation has been rolled out to eligible centres. The GMSR dataset is expandable regarding new research questions and adapts to the specific requests of documenting parties using its dynamic web-based EDC system and modular structure. Physicians schedule frequency of visits according to patients’ needs for medical care and routine follow-ups, which the registry recommends to be carried out at least on an annual basis. Relapses, adverse events, and pregnancies are documented as separate events.

**Table 2:** GMSR dataset.

	Dataset description
Informed consent	Inclusion and exclusion criteria
	Scope of consent
Patient profile	Sex
	Date of birth
	Diagnostic criteria (McDonald, Poser)
	Disease onset
	Date of diagnosis
	Initial symptoms
	Retrospective medical history data
Sociodemographic data	Education
	Occupation
	Family status
	Residence
Disease status	Disease course (lublin categories)
	Expanded disability status scale
	MRI reports
	Multiple sclerosis functional composite-index (Nine-hole peg test, PASAT3, 25-foot walk test)
Symptomatic therapy	Symptoms
	Therapy
Medication^a^	Prior and current disease-modifying treatment
	Treatment duration
	Reasons for treatment discontinuations/switches
(In)dependence	Type of care
	Medical aides
Relapses	Relapse date
	Therapy
Pharmacovigilance-Module*	Comorbidities
	Adverse events
	Pregnancies^a^

*GMSR* German multiple sclerosis registry, *PASAT3* paced auditory serial addition test (3 s-interstimulus interval).

*Voluntary extended documentation, not collected by default.

^a^The GMSR cooperates with the German MS and child wish registry to allow follow up in more detail.

### Data quality

To constantly ensure high data quality, the GMSR established a wide range of quality control measures. The web-based data entry system ensures that centres have the latest versions of questionnaires. Each questionnaire is checked for consistency of dates, of value ranges and for plausibility. Branching logic (asking only relevant questions based on previous answers) ensures that data is only entered for applicable fields. Furthermore, cross-reference-checks compare data between different questions and forms. Longitudinal checks aim at preventing implausible data changes over time. Additionally, the registry uses R-Syntax for automated quality control to find discrepancies and implausibilities. Participating centres receive findings through query management system within the EDC-System. All data changes are recorded in audit trails. The database only accepts records with the completed minimal dataset. Participating centres are provided with training materials and tutorials. Yearly benchmarking reports include visualised comparative data, based on the centre’s population versus other centres’ populations of the same type and individual feedback on data quality. Centres and MSFP can execute ad-hoc statistics and reports as well as customised database searches at any time.

### Data analysis

The GMSR conducts retrospective analysis for a variety of MS-related research questions. These include demographic studies, measurements of care availability, causal inferences but also pharmacovigilance studies and epidemiological assessments^6,22–25^. Most analyses are performed using R^26^. Before data is analysed, it is checked for duplicates, completeness, plausibility and consistency. The GMSR’s data is accessible at reasonable request by any qualified investigator under terms and conditions of the registry’s use-of-access policies and subject to informed consent of patients. Requests for research projects can be proposed via an application form on GMSR’s website^18^.

### Status quo

As of January 2021, 187 centres of all healthcare sectors throughout Germany participate in the GMSR, and the new registry database includes documentation of more than 196,000 consultations of more than 33,000 PwMS. Figure 1 shows the geographic distribution of participating centres across Germany. 5% of registered patients were reported by multiple centres. Recent estimations based on the claims data of participating centres put the number of PwMS treated annually by the participating centres at approximately 90,000. Thus, covering about one-third of the German MS-population (estimated at 240,000–250,000).

**Figure 2:**
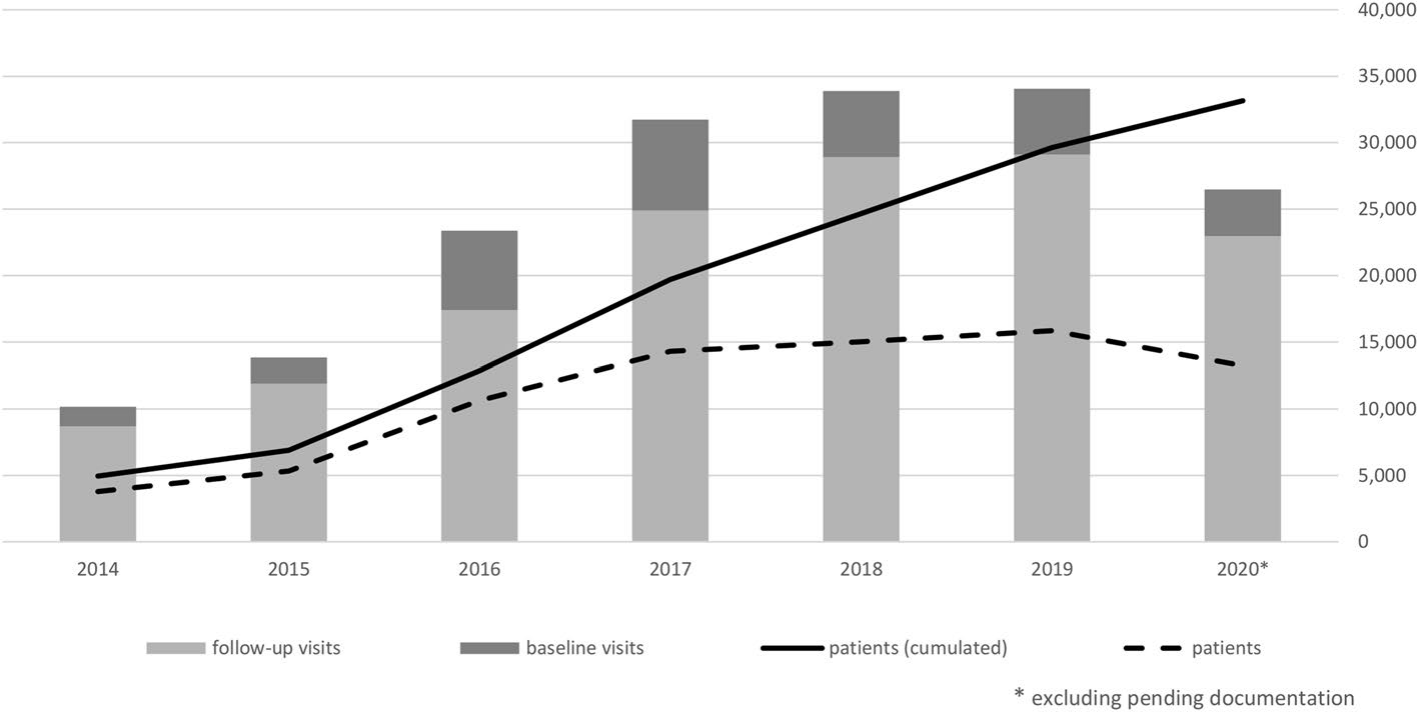
Number of recorded visits per years (by January 2021). Total (cumulative) number of patients included in the GMSR by the end of each year is shown by the black solid line, while the number of patients with actual baseline or follow-up visits during the resp. year is shown by the dashed line. The number of visits per year is show as bars, distinguishing baseline and follow-up visits. *GMSR* German Multiple Sclerosis registry.

At time of data extraction, the yearly documentation for 2020 included 26,491 visits of more than 13,000 patients, with an average of two consultations per patient per year (see Fig. 2). 3530 patients joined the registry in 2020. Table 3 shows the number of visits in which specific (sets of) variables are present.

**Table 3:** Number of patients and visits by scale/category of information.

	Number of visits	Number of patients
Total	196,632	33,174
History of onset and diagnosis (dates)	–	29,019
Education	121,155	27,172
Employment	154,394	28,677
Family	178,794	31,096
Nine-hole peg test	30,019	8194
25-foot walk test	17,829	7808
EDSS	201,712	33,059
MRI	35,427	18,318
DMD (y/n)	164,660	31,734
Current MS-symptoms*	172,032	32,277
(In-)dependence**	165,204	31,597

*DMD* disease modifying drugs, *EDSS* expanded disability status scale, *MRI* magnetic resonance imaging.

*Fatigue, paresis, bowel and bladder disturbances etc.

**Care settings and the prescription/use of aids.

**Figure 3:**
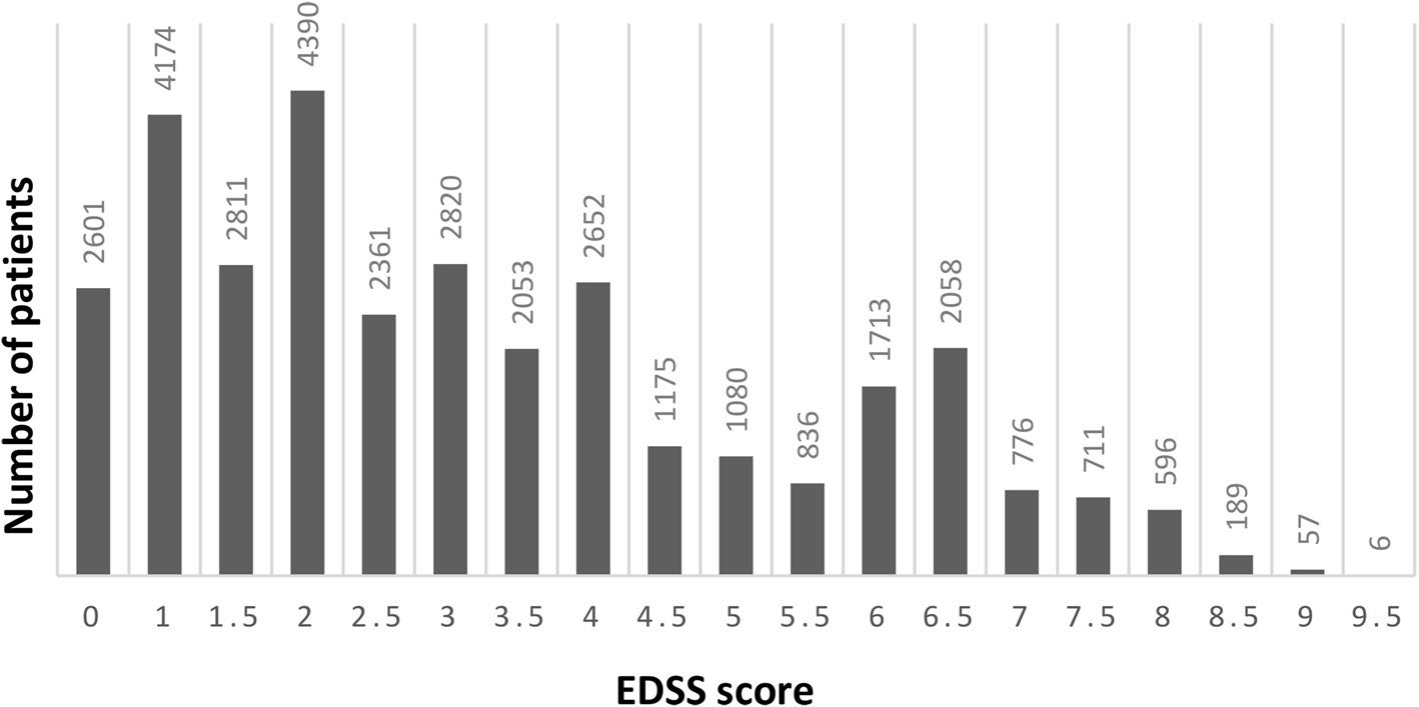
Current EDSS distribution among patients (n = 33,059) in the GMSR. *EDSS* expanded disability status scale, *GMSR* German Multiple Sclerosis registry.

In total, more than 33,000 PwMS were included in the MS Registry since 2014. Baseline data separated by disease course as well as the current distribution of EDSS scores of patients are presented in Supplementary Table S2 and in Fig. 3. In 2020, 4% of visits were documented by rehabilitation centres, 43% by MS centres and 53% by specialised MS centres. By January 2021, 75% of queries on visits data of 2020 were answered and closed. The average time for a final response to a query was 4 (± 7) months (median 1 month) and 81% of all queries were answered.

The GMSR baseline demographic data is comparable to recent publications based on German statutory health claims data regarding female to male ratios, most affected age groups and geographical distribution. The age and disease duration of PwMS at registry entry are presented in Supplementary Figure 1. Compared to European MS registries, there are differences in reported percentages of secondary progressive MS (SPMS) within the registry’s population, whereas the differences in EDSS are narrower. Wider DMT options for patients with relapsing–remitting MS (RRMS) may be the reason for a bias in classification by the treating neurologist^27^. Table 4 compares baseline GMSR patient data with data from the registries of Italy, Denmark, France, and Sweden^28–32^.

**Table 4:** Comparison of patient characteristics.

	German MS registry	Italian MS registry^22^	Danish MS registry^23,24^	OFSEP^25^	Swedish MS registry^26^
Female	71%	67%	69%	71%	70.3%
Age at onset (years)	33.1 ± 10.7	30.5 ± 10.5		32.6 ± 10.8	
CIS	1.8%	5.6%	2.4%	12.1%	
RRMS	75.8%	75.3%	80%	55.2%	67.5%
SPMS	15.7%	12.9%	12.7%	21.4%	24.6%
PPMS	6.7%	6.1%	4.8%	11.2%	7.9%

Patients with unknown disease course are excluded.

*CIS* clinically isolated syndrome, *MS* multiple sclerosis, *OFSEP* observatoire français de la sclérose en plaques, *PPMS* primary progressive MS, *RRMS* relapsing–remitting MS, *SPMS* secondary progressive MS.

### Funding

The establishment and maintenance of the GMSR is funded by the German Multiple Sclerosis Foundation and the German Multiple Sclerosis Society. Additionally, the MSFP has received financial support from Biogen, Bristol Myers Squibb, Merck Serono, Novartis, Roche and Sanofi for the specific purposes of operating the GMSR. Principles of the cooperation between MSFP and pharmaceutical companies are: (1) Transparency: each stakeholder gets the same information at the same time and the type and scope of sponsorship is made public, (2) Financing: to avoid an increased influence of a single stakeholder or a financial dependence on one funder, stakeholders contribute equal amounts, regardless of the company’s market share, (3) Information exchange: stakeholders meet at least once a year to discuss results, ideas for scientific evaluations and further joint activities, (4) Scientific independence: the MSFP has the right to publish registry results independently.

### Registry-based studies and sub-cohorts

Besides the operation of the MS registry, the MSFP develops and provides the EDC system for MS research projects that can (partly) be linked to registry data (see Supplementary Table 2)^33^.

## Discussion

The GMSR is a unique and reliable long-term data repository for real-world data on epidemiology and health of PwMS in Germany. It allows long-term monitoring of the disease course, analysis of different treatments and evaluation of the comparative efficacy, effectiveness and safety of an increasing number of DMT, which cannot be addressed as much by randomised clinical trials (RCT)^10,24,34^. The GMSR holds data on all approved DMT instead of focusing on specific drugs^24^. In RCT, inclusion criteria often do not fit the effect mechanism of the active substance, for instance, patients with low disease activity or long disease duration are included in studies on high anti-inflammatory effect substances^7^. Their high costs and intensity result in their rather short duration and insufficiency concerning real-world effectiveness in the long-term^1,7^. Therefore, registry data is important for patients, physicians, industry, regulatory authorities and health insurance system to provide support to treatment decisions and the development of health promotion measures^5,10^.

The increasing number of DMTs leads to individual treatment decisions, which consider disease related variables like symptoms or clinical findings, but also patient preferences. The evaluation of the severity and significance of specific patient symptoms and their consequences for disease management are often different from that of physicians^35^. Consequently, PRO will become more important^3,7^. The technical infrastructure of the GMSR allows implementation of PRO measurement via eCRFs that can be completed directly by patients and is directly linked to medical data^36^.

The creation of an added value within the GMSR for health professionals to document disease progression and treatment history directly through the EDC system, like automatically generated discharge letters and medical reports, could encourage them to participate in the MS registry and is constantly pursued by the GMSR. A technical interface to the centre’s primary information system could have a similar effect due to the reduced documentation effort. It could furthermore establish a documentation system for several different prospective studies and reduce time and costs expenditure in the future. Despite its positive effects, the reuse and integration of electronic healthcare records for research purposes is still in its early stages^37^.

To increase follow-up rates, it is necessary to provide an incentive for participating centres to document visits regularly. Possible incentives which are offered by the GMSR to achieve this goal are financial compensation, requirements analysis at annual user meetings and guidance documents for follow-up processes.

Over the years, the GMSR’s findings have benefited MS science. The monitoring of PwMS through the registry allowed for the first-time estimates concerning its prevalence, disease types, symptoms and degree of symptomatic treatment. Furthermore, the registry enables register based RCTs and General Data Protection Regulation compliant patient recruitment.

Recently, evaluations of registry data on symptom frequency and symptomatic treatment patterns showed that fatigue is one of the most common symptoms. This has only been analysed in smaller cohorts^38^ but is of high importance as fatigue impacts patients well-being and employment^30^. Through GMSR’s participation in international projects, insights and comparison of data of PwMS from other countries and healthcare systems, such as employment statuses^23^, time until the initiation of a DMT^22^ and validation of secondary progressive MS classification methods^39^ were gained.

However, the GMSR suffers from certain biases and limitations. The registry is not population-based. To date, there is no legal obligation to report MS cases in Germany. The introduction of an obligation would contribute to completeness of the registry’s data and nationwide coverage. PwMS are treated in all areas of care (e.g. general practioners, urologist etc.) and are therefore not always seen regularly in a specialised neurologic unit, thus a legal obligation like in cancer would ensure the completeness. As the registry focuses on neurological care providers of special experience in the treatment of PwMS, the proportion of clinically diagnosed RRMS cases is higher than presented in the German statutory claims data. Our estimations show that since its revision in 2014, the registry has captured more than 33,000 PwMS resulting in about one-third of the PwMS seen in the participating centres. If data from the prior registry data collection, commenced at the turn of the century, is included, the coverage of the GMSR reaches more than 73,000 PwMS. On annual basis the participating specialised centres treat roundabout one-third of the prevalent German MS-population. Especially PwMS suffering from progressive disease courses tend to seek neurological expertise less often, possibly explaining their underrepresentation in the GMSR. However, by including PwMS from specialised rehabilitation centres, the GMSR still captures a higher percentage of the progressive patients than if it would just recruit from outpatient facilities^40^.

Recent research on the conversion of RRMS into SPMS within the GMSR and other registries showed that significant amounts of patients might be clinically labelled as RRMS while still receiving DMTs although objective algorithms would judge the disease course differently^27^.

With legal barriers in place that make the inclusion of under-age PwMS quite challenging and specific paediatric data collections already in place it was decided in the conception phase of the GMSR to allow registry entry only to adult PwMS. Paediatric MS patients are often treated in different settings than adult MS-patients. However, the informed consent allows the collection of retrospective data from patients and paediatric onset MS patients are included after their 18th birthday in the GMSR^41,42^.

With no public funding for maintaining a long-term operation of a data source like the GMSR, funding remains a challenge that cannot be solely covered by the patient society. The GMSR has therefore started to involve the industry for funding. The extension of the dataset by the pharmacovigilance module was an important step for the use of registry data in non-interventional PASS, which was promoted by the EMA^43^. This may reduce costs, increase transparency, and create confidence in the value of pharmacotherapy of MS.

## Supplementary Information

Supplementary Document 1.

Supplementary Table 1.

Supplementary Table 2.

Supplementary Table 3.

Supplementary Figure 1.

Supplementary Information.
