# Supplementary material for: Chances and challenges of a long-term data repository in multiple sclerosis: 20th birthday of the German MS registry
Source: Sci Rep. 2021 Jun 25;11:13340. doi: 10.1038/s41598-021-92722-x (PMC8233364; doi:10.1038/s41598-021-92722-x)
Supplement: Supplementary file 1 — Supplementary Document 1. [file 41598_2021_92722_MOESM1_ESM.docx]

### **Supplementary Document 1: History of GMSR**

In 2001, the German MS Society initiated the establishment of a nationwide MS registry for Germany to collect epidemiological data as well as data on disease course, healthcare, and the social situation of PwMS in a standardised way. For this purpose, the MS Research and Project Development, a non-profit company with limited affairs (MSFP), was founded to establish and manage the GMSR.

During the pilot phase from 2001 to 2003, five centres^[[1]](#footnote-1)^ of different geographical areas and health care sectors that were involved in the treatment of patients with MS participated in the data collection of the initial data set. Patient data was captured via the electronic data capture (EDC) system ‘MSDS Klinik’. The participating centres submitted de-identified medical patient data to the MS registry for merging, quality control, and analysis.(14) In 2005, the German MS Society introduced specific criteria to award medical facilities a certificate as “distinguished MS-centres”. Awarded centres committed themselves to participate in patient recruitment and data collection for the GMSR. During this expansion phase, a network of MS centres was created and allowed the registry to capture data of a comprehensive and representative subset of the German MS population through involvement of all relevant health care sectors that were involved in MS patient care (i.e. university hospitals, neurological clinics, rehabilitations centres and neurological practices).(1)

Until 2009 more than 10.000 PwMS had consented to enter the GMSR and were captured in the registry database. By using hash functions (one-way encryption) for pseudonymisation, double counting of patients was prevented, and longitudinal, cross-centre data collection enabled. At that time, an extensive syntax was developed to identify questionable and implausible records which were then returned to the reporting centre for correction or confirmation. Until correction or confirmation, the data was excluded from analyses.

In 2010, as there was a wider availability of (broadband) internet access in the MS centres, the research database, was implemented as a web-based data capture system using the software secuTrial. In addition, in collaboration with the Competence Network Multiple Sclerosis (KKNMS), a common dataset was developed and implemented.

In the wider context of harmonisation efforts across the German MS community the dataset of the registry was revised in 2014, and the switch to the web-based data capture system was completed. In the same year, patient reported outcome (PRO) measures were additionally integrated.

Until 2018, interfaces to other national (MS) data collections (NeuroTransData^[[2]](#footnote-2)^ [NTD] and REGIMS^[[3]](#footnote-3)^) were implemented. Furthermore, query processes for data cleaning have been expanded.

In 2019, after a pilot phase in 2018, a pharmacovigilance module was finalised, and additional documentation of safety data was made possible on a voluntarily, and for the first time reimbursed, basis. The reimbursement is paid half-yearly to centres which have concluded a supplementary agreement for completely documented baseline or follow-up visits including the additional pharmacovigilance items.

Figure 1: History of the GMSR

*DMSG: German MS Society

**KKNMS: Competence Network Multiple Sclerosis

1. Berlin Jewish Hospital, University Hospital Bochum, Neurological practice Hamburg, Clinic for Neurology of the University of Rostock, Clinic for Neurology of the University Hospital Würzburg [↑](#footnote-ref-1)
2. www.neurotransdata.com [↑](#footnote-ref-2)
3. https://www.medizin.uni-muenster.de/en/epi/research/projects/regims.html [↑](#footnote-ref-3)
