# Supplementary material for: Chances and challenges of a long-term data repository in multiple sclerosis: 20th birthday of the German MS registry
Source: Sci Rep. 2021 Jun 25;11:13340. doi: 10.1038/s41598-021-92722-x (PMC8233364; doi:10.1038/s41598-021-92722-x)
Supplement: Supplementary file 2 — Supplementary Table 1. [file 41598_2021_92722_MOESM2_ESM.docx]

**Supplementary Table 1: Minimal Dataset of GMSR**

| **Minimal Dataset of the German MS Registry** | | |
| --- | --- | --- |
| ***Entry visit*** | Informed consent | consent status  inclusion criteria  exclusion criteria |
|  | Patient profile | sex  date of birth  diagnostic criteria (McDonald, Poser)  type of disease course (ROMS/POMS)  date of onset of disease  date of diagnosis  initial symptoms |
|  | Sociodemographic data | school graduation  occupational education  occupation  employment  marital status  home support  state of residence |
|  | Disease status | current disease course  EDSS  MRI  relapses  MSFC (9 Hole-Peg-Test, PASAT3, 25ft-Walkingtest)  current symptoms and symptomatic treatment  disease modifying treatment |
|  | (In)dependence | type of care  medical aids |
| ***Follow-up vis*it** | Changes in sociodemographic data |  |
|  | Disease status | current disease course  EDSS  MRI  relapses  MSFC (9Hole-Peg-Test, PASAT3, 25ft-Walkingtest)  current symptoms and symptomatic treatment |
|  | Changes in care situation |  |

*EDSS, expanded disability status scale; MRI, magnetic resonance imaging; MS, multiple sclerosis; MSFC, multiple sclerosis functional composite; PASAT3, paced auditory serial addition test (3 seconds-interstimulus interval); POMS, progressive-onset MS; ROMS, relapsing-onset MS.*
