# Supplementary material for: Chances and challenges of a long-term data repository in multiple sclerosis: 20th birthday of the German MS registry
Source: Sci Rep. 2021 Jun 25;11:13340. doi: 10.1038/s41598-021-92722-x (PMC8233364; doi:10.1038/s41598-021-92722-x)
Supplement: Supplementary file 3 — Supplementary Table 2. [file 41598_2021_92722_MOESM3_ESM.docx]

**Supplementary Table 2: Baseline Data stratified by current disease course.**

| **Patient Characteristics** | | | | | |
| --- | --- | --- | --- | --- | --- |
|  | **All patients** | **CIS** | **RRMS** | **SPMS** | **PPMS** |
| ***n  (%)*** | 33174 | 562 (1.7%) | 24052 (72.5%) | 4977 (15.0%) | 2128  (6.4%) |
| ***female (%)*** | 71.3% | 68.5% | 73.1% | 69.1% | 56.4% |
| ***age onset, current (years)***  ***[mean, SD]*** | 33.1±10.7 | 36.5±11.0 | 32.2±10.2 | 33.3±10.5 | 42.8±10.9 |
| ***disease duration, last visit (years)***  ***[mean, SD]*** | 13.7±10.1 | 4.0±6.2 | 12.0±8.9 | 23.6±10.3 | 14.5±10.1 |
| ***EDSS-score [median, Q25, Q75]*** | 3.0 (1.5, 4.5) | 1.5 (1.0, 2.0) | 2.0 (1.0, 3.5) | 6.0 (4.5, 7.0) | 5.5 (3.5, 6.5) |
| ***Diagnosis delay (years) [mean, SD]*** | 1.7±4.0 | ±0.4±1.4 | 1.5±3.6 | 3.0±5.8 | 2.7±4.8 |

*CIS, clinically isolated syndrome; EDSS, expanded disability status scale; MS, multiple sclerosis; n, number of patients; PPMS, primary progressive MS; RRMS, relapsing-remitting MS; SD, standard deviation; SPMS, secondary progressive MS.*
