# Supplementary material for: Chances and challenges of a long-term data repository in multiple sclerosis: 20th birthday of the German MS registry
Source: Sci Rep. 2021 Jun 25;11:13340. doi: 10.1038/s41598-021-92722-x (PMC8233364; doi:10.1038/s41598-021-92722-x)
Supplement: Supplementary file 4 — Supplementary Table 3. [file 41598_2021_92722_MOESM4_ESM.docx]

**Supplementary Table 3: Registry-based studies and sub-cohorts within the GMSR.**

| EmBioProMS | Since 2018, the MSFP is providing the EDC system for an explorative study of emerging blood biomarkers in progressive multiple sclerosis (EmBioProMS) that is funded by the German Multiple Sclerosis Society. Four university hospitals and two specialised clinics are recording study data that can be linked to MS Registry data via Patient-ID if the patient is participating in both projects. (30) |
| --- | --- |
| Power@MS | Within the Power@MS Project funded by the innovations fond of the German Federal Joint Committee and led by the university hospital Hamburg-Eppendorf, MSFP is implementing the documentation platform for two registry-based randomised controlled trials aiming to strengthen the empowerment of PwMS. Data that are captured for this project are directly linked to Registry data. (31) |
| MS-registry for children in NRW | For a pilot-project on epidemiology and healthcare research on paediatric patients with MS in cooperation with Vestische Kinder- und Jugendklinik Datteln of Witten/Herdecke University, the MSFP is has established the database and is providing support. |

*GMSR, German Multiple Sclerosis Registry.*
