# Supplementary material for: Chances and challenges of a long-term data repository in multiple sclerosis: 20th birthday of the German MS registry
Source: Sci Rep. 2021 Jun 25;11:13340. doi: 10.1038/s41598-021-92722-x (PMC8233364; doi:10.1038/s41598-021-92722-x)
Supplement: Supplementary file 5 — Supplementary Figure 1. [file 41598_2021_92722_MOESM5_ESM.docx]

**Supplementary Figure 1: Entry into GMSR by age and disease duration**


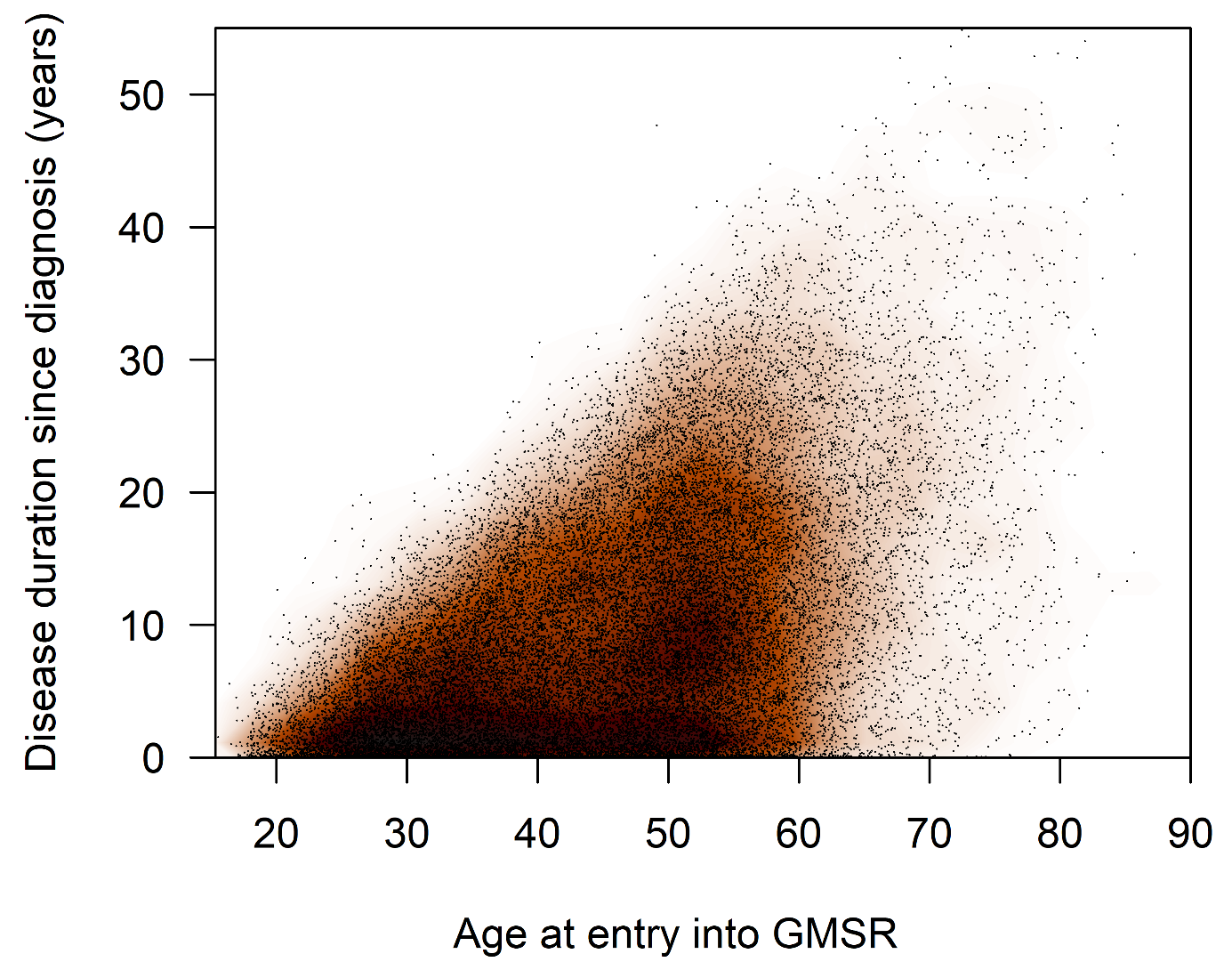


Scatterplot of age (x-axis) and disease duration (y-axis) of patients when entering the GMSR. A two-dimensional density estimate is plotted as a heatmap overlay (orange shaded area). An axis-aligned bivariate normal kernel was used. Lower case numbers enter the GMSR above the age of 65 years. GMSR, German Multiple Sclerosis Registry.
