# Supplementary material for: Chances and challenges of a long-term data repository in multiple sclerosis: 20th birthday of the German MS registry
Source: Sci Rep. 2021 Jun 25;11:13340. doi: 10.1038/s41598-021-92722-x (PMC8233364; doi:10.1038/s41598-021-92722-x)
Supplement: Supplementary file 6 — Supplementary Information. [file 41598_2021_92722_MOESM6_ESM.docx]

**Acknowledgments**

We would like to thank all patients that have given their informed consent to be included the GMSR. Furthermore, our thanks go out to all members of staff in all contributing centers for their continued efforts. We would like to thank the following centers for patient recruitment and data collection:

Medizinische Versorgungszentren, Altenburger Land GmbH, Altenburg

Gemeinschaftspraxis Dr. med. A. Safavi und M. Schädel, Fachärzte für Neurologie, Alzenau

Neurozentrum Alzey, Dr. Loos / Dr. Bernhard, Alzey

Rhein-Mosel-Fachklinik Andernach, Zentrum für Psychiatrie, Psychotherapie und Neurologie, Andernach

DRK Kamillus Klinik, Neurologische Abteilung, Asbach

Praxis Dr. Hofmann, Aschaffenburg

Zentrum für Neurologie, Psychiatrie und Psychotherapie Asperg, Asperg

HELIOS Klinikum Aue, Klinik für Neurologie, Aue

Universitätsklinikum Augsburg, Neurologische Klinik mit klinischer Neurophysiologie, Augsburg

Praxis für Neurologie, Psychiatrie & Psychotherapie, Dr. Schöll, Dr. Steidl & Kollegen, Bad Homburg

August-Bier-Klinik, Fachklinik für Neurologie, Neurotraumatologie und Rehabilitation, Bad Malente-Gremsmühlen

Caritas-Krankenhaus Bad Mergentheim gGmbH, Klinik für Neurologie, Bad Mergentheim

Rhön-Klinikum Campus Bad Neustadt, Klinik für Neurologie, Bad Neustadt a. d. Saale

Johanniter Ordenshäuser, Neurologische Abteilung, Bad Oeynhausen

MediClin Reha-Zentrum Bad Orb, Neuro-orthopädisches Reha-Zentrum, Bad Orb

Agaplesion Ev. Bathildis Krankenhaus, Neurologische Ambulanz, Bad Pyrmont

Segeberger Kliniken GmbH, Neurologisches Zentrum, Bad Segeberg

Neurologisches Rehabilitationszentrum Quellenhof, Bad Wildbad

Wicker-Klinik, Neurologische Abteilung, Bad Wildungen

Dr. Becker Kiliani-Klinik, Neurologische Abteilung, Bad Windsheim

Hardtwaldklinik I, Neurologisches Zentrum, Werner Wicker GmbH & Co. KG, Bad Zwesten

Neurologische Klinik Selzer GmbH, Baiersbronn

Kallmann Neurologie - Multiple Sklerose Zentrum Bamberg (MSZB), Dr. med. Boris-A. Kallmann, Bamberg

Klinik Hohe Warte Bayreuth, Neurologische Klinik, Bayreuth

Marianne-Strauß-Klinik, Behandlungszentrum Kempfenhausen für Multiple Sklerose Kranke gemeinnützige GmbH, Berg

Charité - Universitätsmedizin Berlin, Ambulanz für Multiple Sklerose und Neuroimmunologie, Berlin

Jüdisches Krankenhaus Berlin, Akademisches Lehrkrankenhaus der Charite, Zentrum für Multiple Sklerose, Berlin

Alexianer St. Joseph-Krankenhaus Berlin-Weißensee, Klinik für Neurologie / MS Ambulanz §116 B, Berlin

Vivantes Klinikum Neukölln - Klinik für Neurologie, Spezialambulanz für Multiple Sklerose, Berlin

Neurologische Praxis, MD Turki Akil, Berlin

Evangelisches Krankenhaus Bethel gGmbH, Klinik für Neurologie, Bielefeld

Neurozentrum Bielefeld-Brackwede, Dres. med. J. und M. Böhringer, Dr. J. Katzmann, F. Sudfeldt, Bielefeld

Medical Park Loipl, Bischofswiesen

Neurologische Praxis, Böblingen

St. Josef-Hospital, Klinikum der Ruhr-Universität Bochum, Klinik für Neurologie, Bochum

Gemeinschaftspraxis Dres. med. Niederhofer, Kauermann, Küper, Bochum

Berufsgenossenschaftliches Universitätsklinikum Bergmannsheil gGmbH, Neurologische Klinik und Poliklinik, Bochum

Neurologische Facharztpraxis Dr. I. Nastos, Bochum

Gemeinschaftspraxis Kausch & Lippert, Bogen

Neurologisches Rehabilitationszentrum Godeshöhe e.V., Bonn

Neurologische Gemeinschaftspraxis, Bonn

Asklepios Fachklinikum Brandenburg, Klinik für Neurologie, Brandenburg

Neurozentrum Schlosscarree, Dr. med. Ekkehard Klippel, Braunschweig

Neurologie am Ziegenmarkt, Braunschweig

Städtisches Klinikum Braunschweig gGmbH, Neurologische Klinik, Braunschweig

Klinikum Bremerhaven Reinkenheide, Neurologische Tagesklinik, Bremerhaven

Carl-Thiem Klinikum Cottbus, Klinik für Neurologie, Cottbus

Dr. med. Martin Delf, Facharzt für Neurologie, Dahlwitz-Hoppegarten bei Berlin

VAMED Rehaklinik Damp, Abteilung Neurologie, Damp

Bezirksklinikum Mainkofen, Neurologische Klinik, Deggendorf

Neurologische Gemeinschaftspraxis Dillingen, Dillingen

Praxis für Neurologie Leclaire/Rotermund, DOC Center Dortmund, Dortmund

Multiple Sklerose Zentrum Dresden, Neurologische Uniklinik Dresden, Dresden

Christophorus - Kliniken GmbH, Neurologische Klinik Dülmen, Dülmen

Medizinisches Versorgungszentrum Düren - Lendersdorf, Praxis Dr. Brand, Düren

Heinrich-Heine-Universität Düsseldorf, Westdeutsches MS-Zentrum Düsseldorf, Düsseldorf

Martin Gropius Krankenhaus Eberswalde, Klinik für Neurologie, Eberswalde

Neurologie im MVZ und Klinikum Eisenach, Eisenach

Praxis Dr. Kirchhöfer, Erfurt

HELIOS Klinikum Erfurt GmbH, Klinik Für Neurologie, Erfurt

Universitätsklinikum Erlangen, Klinik für Neurologie, Erlangen

Dr. med. Edgar Bollensen, Facharzt für Neurologie und Psychiatrie, Eschwege

Universitätsklinikum Essen, Klinik für Neurologie, MS-Ambulanz, Essen

Alfried-Krupp-Krankenhaus, Klinik für Neurologie, Essen

Zentrum für ambulante Neurologie, Essen

Praxis Nervenstark, Essen

Fachklinik Feldberg GmbH, Klinik am Haussee, Feldberger Seenlandschaft

Krankenhaus Nordwest GmbH, Neurologische Klinik, Frankfurt/Main

Universitätsklinikum Frankfurt, Klinik für Neurologie, Frankfurt/Main

Kliniken Schmieder Gailingen, Neurologisches Fach- und Rehabilitationskrankenhaus, Gailingen

MVZ Gelderland, Dr. med. Peter Asmus, Geldern

SRH Waldklinikum Gera GmbH, Klinik für Neurologie, Gera

Gemeinschaftspraxis Dres. Ivancic & Kollegen, Gersthofen

Kath. Kliniken Emscher-Lippe GmbH, St. Barbara-Hospital, Neurologische Abteilung, Gladbeck

Dr. med. Kornelia Seidel, Praxis für Neurologie und Psychiatrie, Gladenbach

Universitätsmedizin Göttingen, Klinik für Neurologie, Göttingen

Universitätsmedizin Greifswald, Klinik und Poliklinik für Neurologie, Greifswald

VAMED Klinik Hagen-Ambrock GmbH, Klinik für Neurologie, Hagen

Städtisches Krankenhaus Martha-Maria Halle-Dölau gGmbH, Klinik für Neurologie, Halle

Lars Daume, Facharzt für Neurologie, Halle

Praxis für Neurologie und Psychiatrie, Dr. med. Birte Elias-Hamp, Hamburg

Neurozentrum Eidelstedter Höfe, Hamburg

RehaCentrum Hamburg GmbH, Fachbereich Neurologie, Hamburg

Universitätsklinikum Hamburg-Eppendorf, Institut für Neuroimmunologie und Multiple Sklerose (INIMS), Hamburg

Klinikum Hanau GmbH, Klinik für Neurologie, Hanau

Universitätsklinikum Heidelberg, Neurologische Klinik, Heidelberg

Medizinisches Versorgungszentrum, Zentrum für Sozialpsychiatrie und Nervenheilkunde am Ostebogen GmbH, Hemmoor

Klinik Hennigsdorf, Oberhavel Kliniken GmbH, Neurologische Abteilung, Hennigsdorf

Gemeinschaftskrankenhaus Herdecke, Klinik für Neurologie, Herdecke

Klinikum Herford, Klinik für Neurologie / MS-Ambulanz, Herford

Medizinisches Versorgungszentrum Herne, Herne

Haus- und Facharztzentrum Laucherttal/Alb, Praxis für Neurologie, Hettingen

Celenus Klinik für Neurologie Hilchenbach, Hilchenbach

HELIOS Fachkliniken Hildburghausen, Klinik für Neurologie, Hildburghausen

Praxis für Neurologie, Doctor-medic Claudia Man, Höxter

Klinikum Ibbenbüren, Klinik für Neurologie, Ibbenbüren

MVZ Immenstadt Allgäu GmbH, Neurologie, Psychiatrie, Psychotherapie, Immenstadt

Alexianer Misericordia GmbH - Augustahospital Anholt, Klinik für Neurologie, Isselburg-Anholt

Neurozentrum am Klosterforst, Itzehoe

Universitätsklinikum Jena, Neurologische Klinik, Jena

ZNS Kamen, Neurologisch-Psychiatrische Praxisgemeinschaft, Kamen

Städtisches Klinikum Karlsruhe gGmbH, Neurologische Klinik -Haus D-, Karlsruhe

Neurologische Gemeinschaftspraxis Kassel Vellmar, Lassek / Dr. Ammerbach / Dr. Fetzer / Fischer, Kassel

Universitätsklinikum Schleswig-Holstein, Ambulanz und Tagesklinik für Neuroimmunologie und Multiple Sklerose, Kiel

Heilig Geist-Krankenhaus, Klinik für Neurologie, Köln

Universitätsklinikum Köln, Klinik und Poliklinik für Neurologie, Köln

Praxis rechts vom Rhein, Dr. med. Jan-Dirk Seifert und PD Dr. med. Hela-Felicitas Petereit, Köln

NeuroMed Campus Nelles, Scharpegge, Haupt, Scharwat, Fachärzte für Neurologie, Schmerztherapie, Köln

Kliniken Schmieder Konstanz, Neurologisches Fach- und Rehabilitationskrankenhaus, Konstanz

Facharztpraxis für Neurologie und Psychiatrie, im Facharztzentrum am Klinikum Konstanz, Konstanz

Praxisgemeinschaft Neurologie am Zoo, Krefeld

Gemeinschaftspraxis Dr. med. B. Wittmann & P. Rieger, Landshut

Praxis Dr. Pfeffer & Dr. Staudinger-Pfeffer, Praxis für Neurologie und Psychiatrie, Landshut

hygieia.net Leipzig, Leipzig

Ärztehaus Stötteritz, Neurologische Praxis, Leipzig

Klinikum Lippe-Lemgo, Neurologische Klinik, MS-Ambulanz Klinikum Lippe Lemgo, Lemgo

Helios Klinik Lengerich GmbH, Abteilung Neurologie, Lengerich

Märkische Kliniken GmbH, Klinikum Lüdenscheid, Klinik für Neurologie, Lüdenscheid

Neuropsychiatricum, Dr. Deibel / Dr. Fischer / Dr. Klenk / Dr. Kohlmaier / Dr. Stenzel, Ludwigshafen

St.-Marien-Hospital GmbH, Neurologische Klinik, Lünen

Gemeinschaftspraxis Katte, Vogelsang-Dietz, Graf, Lünen

Neurologische Praxis U. Kullik, Lutherstadt Eisleben

MS-Spezialambulanz Stephanik, Universitätsklinikum Magdeburg, Magdeburg

MEDIAN Klinik NRZ Magdeburg, Magdeburg

Universitätsklinikum Marburg, Klinik für Neurologie, Marburg

Neurologie in Meerbusch, Meerbusch

Gesundheitszentrum Glantal, Meisenheim

Neurologische Gemeinschaftspraxis, Memmingen, Memmingen

Neurologische Gemeinschaftspraxis im medicentrum Mönchengladbach, Mönchengladbach

Fachübergreifende Gemeinschaftspraxis Neurologie & Radiologie, Mosbach

Ökumenisches Hainich Klinikum gGmbH, Klinik für Neurologie, Mühlhausen

Neuro-Psychiatrisches Zentrum Riem, München

Gemeinschaftspraxis Rickert / Enck / Jansen, Münster

Herz-Jesu-Krankenhaus Hiltrup GmbH, Klinik für Neurologie mit klinischer Neurophysiologie, Münster

Neurologie am Ludgeriplatz, Münster

Gemeinschaftspraxis Dres. Wiborg, Kramer, Brummer, Neu-Ulm

Praxis Dr. Bergmann & Kollegen, Praxis für Neurologie und Psychotherapie, Neuburg

Praxis Berkenfeld, Neukirchen-Vluyn

Ruppiner Kliniken GmbH, Klinik für Neurologie, MS-Ambulanz, Neuruppin

Gemeinschaftspraxis Dr. Rieth, Saur, Dr. Pfister, Neusäß

Nervenärztliche Gemeinschaftspraxis Nürnberg, Nürnberg

Gemeinschaftspraxis Dres. Uhlig/Windsheimer, Nürnberg

Praxis Dres. Schlüter, Beckmann, Berufsausübungsgemeinschaft, Öhringen

MVZ für Neurologie der Paracelsus-Klinik Osnabrück, Osnabrück

Neurologie an der Hase, Holger Lorenzen, Dr. med. Edzard Ites, Stephan Ulf Sylvester, Jens Gläscher, Dr. med. Elisabeth Rehkopf, Osnabrück

Klinikum Osnabrück GmbH, Neurologische Klinik, Osnabrück

Praxis Dr. med. Christoph Schenk, Osnabrück

MVZ Dr. Roth & Kollegen GbR, Facharzt für Nervenheilkunde, Ostfildern

St. Vincenz-Krankenhaus GmbH Paderborn, Neurologische Klinik, Paderborn

Neurozentrum Peine, Peine

Facharztpraxis für Neurologie und Psychiatrie, Andreas Stockert und Dr. Claudia Rettenmayr, Pforzheim

St. Josefs-Krankenhaus Potsdam, Klinik für Neurologie, Potsdam

Praxis Dr. Altmann, Facharzt für Neurologie und Psychiatrie, Potsdam

VAMED Klinik Schloss Pulsnitz GmbH, Pulsnitz

Neurozentrum Ravensburg, Dres. Dieterle / Kunz, Ravensburg

Neurologische Praxis, Maier-Janson / Friedrich, Ravensburg

Knappschaftskrankenhaus Recklinghausen, Klinik für Neurologie und Klinische Neurophysiologie, Recklinghausen

Neurologische Praxis Dr. Wendelin Kyrill Blersch, Regensburg

Universitätsklinikum Regensburg, Klinik und Poliklinik für Neurologie am Bezirksklinikum Regensburg, Regensburg

Sächsisches Krankenhaus f. Psychiatrie u. Neurologie, Zentrum für Psychiatrie, Psychotherapie, Psychosomatik und Neurologie, Rodewisch

Universitätsmedizin Rostock, Klinik und Poliklinik für Neurologie, Rostock

Immanuel-Klinik-Rüdersdorf, MS-Ambulanz, Rüdersdorf

ZNS Südpfalz - Zentrum für Nervensystem & Seele, Rülzheim

Nordwest-Krankenhaus Sanderbusch, Neurologie, Sande

Medizinisches Versorgungszentrum Schlüchtern, Schlüchtern

Diakonie-Klinikum Schwäbisch Hall gGmbH, Neurologische Klinik, MS-Ambulanz, Schwäbisch Hall

Hephata-Klinik, Neurologie, Schwalmstadt

Fachklinik für Neurologie Dietenbronn GmbH, Akademisches Krankenhaus der Universität Ulm, Schwendi

HELIOS Kliniken Schwerin, Neurologische Klinik, Schwerin

Berufsausübungsgemeinschaft Prof. Wagner & Dr. Kaltenmaier, Schwetzingen

Asklepios Kliniken Schildautal Seesen, Klinik für Neurologische Rehabilitation, Seesen

Asklepios Kliniken Schildautal, Klinik für Neurologie, Seesen

Neurologisch-Psychiatrische Gemeinschaftspraxis, Dr. Schulz, Dr. Lindemuth, Hübner, Dr. Heimel, Albertus Magnus Zentrum, Siegen

Neurologische Praxis, MVZ Siegerlandzentrum, Siegen

E/M/S/A, Zentrum für Neurologie / Psychiatrie / Neuroradiologie, Singen

MediClin Klinikum Soltau, Soltau

Neurologische Praxisgemeinschaft, Sprockhövel

MediClin Bosenberg Kliniken, St. Wendel

Asklepios Fachklinikum Stadtroda, Klinik für Neurologie, Stadtroda

ZNS Straubing, Straubing

Klinikum Stuttgart Katharinenhospital, Neurologische Klinik, Neurozentrum, Stuttgart

mind mvz GmbH, Stuttgart

Neurozentrum Sophienstrasse, Dr. Herbst, Dr. Wannenmacher, Dr. Hartmann, Stuttgart

Neurologisch-Psychiatrische Praxis Dr. Susanne Weber, Stuttgart

Neurologische und psychiatrische Praxis Dr. Kowalik, Stuttgart

Dres. med. M. Appy, W. Molt, Prof. A. Melms & Kollegen, Berufsausübungsgemeinschaft, Stuttgart

Knappschaftskrankenhaus Sulzbach, Neurologische Klinik, Sulzbach / Saar

Sauerlandklinik Hachen, Neurologische Spezialklinik Multiple Sklerose, Sundern

Asklepios Fachklinikum Teupitz, Neurologische Klinik, Teupitz

Krankenhaus der Barmherzigen Brüder, Abteilung für Neurologie und Neurophysiologie, Trier

Behandlungszentrum für Multiple Sklerose, GFO Kliniken Troisdorf, Troisdorf

Universitätsklinikum Tübingen, Zentrum für Neurologie, Tübingen

Helios Klinikum Uelzen GmbH, Uelzen

Universitätsklinikum Ulm, Klinik und Poliklinik für Neurologie, Ulm

Neuropraxis München Süd, Unterhaching

Westerwaldklinik Waldbreitbach gGmbH, Rehabilitationszentrum für Neurologie und Neurologische Psychosomatik, Waldbreitbach

Dr. med. Sabine Gschrey & Dr. med. Gerhard Gschrey, Ärzte für Neurologie und Psychiatrie, Wendlingen

Klinikum St. Georg / Fachkrankenhaus Hubertusburg, Klinik für Neurologie und Neurologische Intensivmedizin, Wermsdorf

Gemeinschaftspraxis Dr. Springub / Schwarz, Westerstede

Ammerland-Klinik GmbH, Klinik für Neurologie, Westerstede

DKD HELIOS Klinik Wiesbaden, Fachbereich Neurologie, Wiesbaden

MEDIAN Klinik Wilhelmshaven, Fachklinik für Orthopädie und Neurologie, Wilhelmshaven

Neurologisch-Psychiatrische Gemeinschaftspraxis Wolfenbüttel, Wolfenbüttel

Neurologische Praxis am Klieversberg, Wolfsburg

Praxis Dr. med. J. D. Seybold, Würzburg

Universitätsklinikum Würzburg, Neurologische Klinik und Poliklinik, Würzburg
